# Boosting health skills in Ukraine

**DOI:** 10.2471/BLT.15.020815

**Published:** 2015-08-01

## Abstract

Health workers in conflict-hit eastern Ukraine are gaining new skills that may prove invaluable in peacetime too. Andrei Shukshin reports.

For more than a year Ukraine has been torn asunder by a bitter conflict that has triggered a growing humanitarian crisis.

But amidst the chaos, the country has one important asset: human capital.

“The situation is very difficult and it’s thanks to our health workers that this country’s health-care system is still running,” says Dr Tatiana Dubinina, chief medical officer at the Ukrainian health ministry’s Department of Medical Assistance to Children.

“Since conflict erupted in the eastern regions, our doctors and nurses have been working with utmost dedication.”

“It’s thanks to our health workers that this country’s health-care system is still running.”

Tatiana Dubinina

In spite of truce agreements last September and in February of this year, fighting has continued in some places and the hostilities have taken a dramatic toll on the region: some 1.3 million people have been displaced and many local health workers have been wounded, killed or have fled.

Medics, emergency responders and volunteers have been vital in keeping Ukraine’s already overstretched and inefficient health-care system going.

Dr Petro Ishchenko, head of the paediatric care department at the Children’s Hospital in the eastern city of Lysychansk, vividly remembers the battles on 21 July last year when his wards began filling up with wounded people.

Ishchenko, who as duty doctor ran the hospital throughout the week of heavy fighting that resulted in hundreds of casualties, told his staff they were free to go, but only a few fled the violence.

“There was no power, no water, no public transport, so doctors and nurses had to walk or cycle to work, often dodging the bullets,” Ishchenko says. “I have a picture of our children’s surgeon taking a break after performing several emergency amputations in a row – his overalls covered in blood. He hates this picture because no doctor would want to look like that. But that’s how it was.”

The government has been increasing the number of emergency response teams, boosting stocks of medicines and other medical supplies as well as setting up air-raid shelters in hospitals and a system of medical service provision for the internally displaced.

“The medical staff in the affected areas carried out these tasks without delay as they felt a great sense of responsibility to the people,” says Dubinina.

The World Health Organization (WHO) has been working closely with Ukraine’s health ministry on the emergency health response and, since the conflict began, has sent many convoys of medical supplies to the areas where health facilities have been destroyed or damaged and the health needs of the population are great.

“There are shortages of vaccines, medicines and medical supplies and an increased need for health care compounded by increased deprivation,” says Dr Dorit Nitzan, WHO Representative in Ukraine.

“As the United Nations health agency, WHO is leading the United Nations’ Health and Nutrition cluster in the Ukraine emergency,” Nitzan says, adding: “As such, WHO responded straightaway by setting up a network of mobile units and posts providing emergency primary health-care services.”

The mobile units and posts have been up and running since February and are funded by the European Union, the United Nations’ Central Emergency Response Fund and other donors, including Canada, Finland, Israel and the United Kingdom of Great Britain and Northern Ireland.

Boosting the skills of the people in Ukraine who are providing both emergency and routine health-care services has been key to improving the health response.

WHO is helping to build capacity in Ukraine in several ways.

Treatment protocols, standard operating procedures and decision trees have been integrated into a new WHO Ukraine tablet application for staff working in the emergency health response. WHO has been working with the Ukraine health ministry to redefine the role of nurses as modern health professionals in their own right and not merely supporting physicians. And WHO has been providing training for health workers.

“When there’s fighting all around people tend to focus on trauma, which is understandable,” says Ishchenko. “But we shouldn’t forget that people still suffer and die if not treated promptly and properly for illnesses or conditions unrelated to the violence.”

“At times like this it is vital to have qualified personnel in place, but doctors can’t do everything. You need nurses and orderlies to stand in and perform some tasks,” Ishchenko says.

Ishchenko was one of 35 Ukrainian physicians who attended a workshop on WHO’s emergency triage assessment and treatment (ETAT) guidelines in the Ukrainian capital of Kyiv in March, given by WHO experts and trainers from Armenia, Kazakhstan and the Republic of Moldova.

The ETAT course teaches health workers to triage sick children when they arrive at a hospital or mobile health unit and provide emergency treatment for life-threatening conditions.

The ETAT guidelines were originally developed for low- and middle-income countries, where health facilities are not set up to handle large numbers of patients arriving at the same time and to identify and treat patients with the most serious conditions first.

The guidelines were adapted earlier this year to Ukraine’s situation by omitting sections on the management of malnutrition and severe dehydration in children, and condensing the rest. These were then translated into Russian and Ukrainian.

After the workshop in Kyiv, Ishchenko and his colleagues returned to their hospitals in Dnepropetrovsk, Zaporozhye, Kharkiv and Kyiv provinces, passing on their newly acquired knowledge and skills to colleagues working in mobile units and emergency medical services; nurses working on admission desks, and emergency-room teams in Ukraine’s eastern regions.

The ETAT training involves learning a set of simple algorithms to decide which children to treat first and to treat shock, coma, convulsion and blocked airways on the spot. “The training has helped to boost participants’ general skills and their level of preparedness for such emergencies,” says Dr Martin Weber, a programme manager for child and adolescent health in the WHO Regional Office for Europe who helped to adapt the ETAT guidelines.

Not everyone embraced the idea of applying the ETAT guidelines to the situation in Ukraine at first. Some officials and doctors questioned the relevance of a triage course designed for developing countries.

But since the first workshop in March, support – and demand for the knowledge and skills – has grown.

“Along with those formally enrolled on the course, many people – mostly from the emergency health services – approached us asking if they could attend as well,” Ishchenko says. “They weren’t interested in certificates, they just wanted to learn how to handle situations when children are brought to them in distress.”

For Dr Ivan Anikin, head of the newborn intensive care department at the Zaporozhye Regional Children’s Hospital, the Ukrainian health authorities’ support for the ETAT training programme has been an important element of the emergency response.

“Managing the flows of patients in critical condition in areas affected by the fighting has been a major focus of attention of Ukraine’s health authorities. Health authority efforts combined with training carried out by WHO and the health ministry made it possible to bring the number of cases of delayed health assistance to children to [close to] zero,” Anikin says.

Ishchenko has since given an ETAT workshop under WHO expert supervision in the city of Kharkiv and trained staff at his hospital in Lysychansk in the approach.

“The beauty of this approach is that it doesn’t require gadgets or gizmos. It is simple and straightforward. We need people with this training and we need them fast. The WHO ETAT provides an effective way to achieve that,” he says.

“The WHO launched ETAT here to help Ukraine deal with the crisis. But … we need it just as much in peacetime.”

Petro Ishchenko

“Our health-care system is in decline. The crisis has just exposed the deficiencies that had been papered over so carefully for so long,” Ishchenko says. “The WHO launched ETAT here to help Ukraine deal with the crisis. But it goes far beyond that. We need it just as much in peacetime”.

**Figure:**
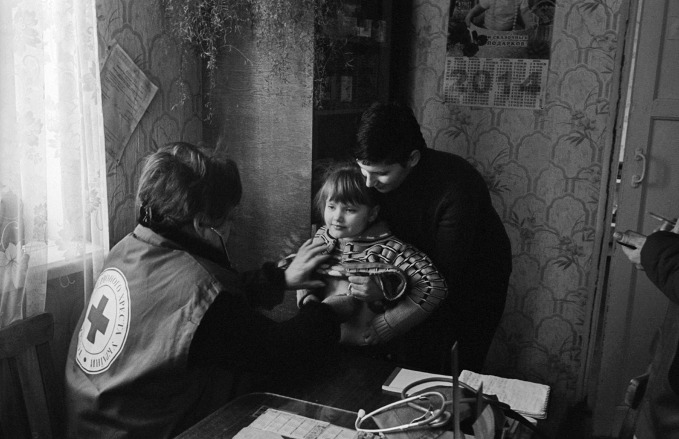
Health workers in eastern Ukraine provide a girl and her mother with a routine health check-up.

WHO/Alex Glyadyelov

**Figure:**
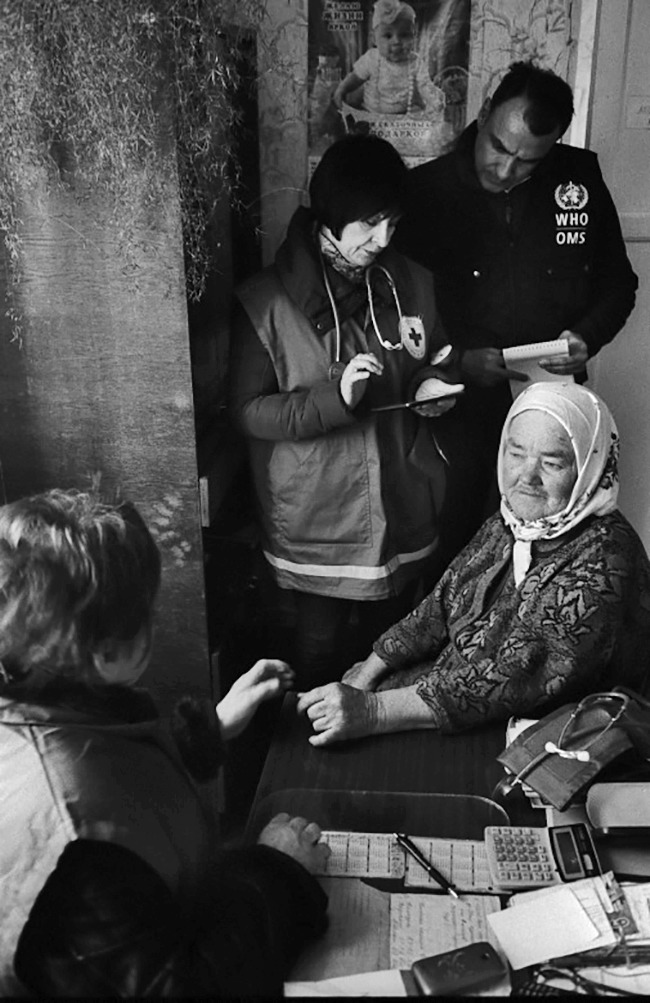
This 70-year-old woman, who was relocated from her home in Donetsk, is being examined in the Kharkiv region by a health worker from the Ukrainian Red Cross.

WHO/Alex Glyadyelov

